# Supplementary material for: Content-rich biological network constructed by mining PubMed abstracts
Source: BMC Bioinformatics. 2004 Oct 8;5:147. doi: 10.1186/1471-2105-5-147 (PMC528731; doi:10.1186/1471-2105-5-147)
Supplement: Additional File 2 — The original results of the above study (non-essential files are deleted to keep the file size under the limit set by BMC bioinformatics). [file 1471-2105-5-147-S2.bz2 › chilibotAdditionalFile2/dip20/41ID7545910E165/html/TBP_TAF40.html]

 


 **TBP** and **TAF40** 
  
Found 14 abstracts in PubMed, retrieved 14.  
 

 What does Google say? 
 PDF only 
| .edu only 

---

**Interactive relationship** (e.g. stimulation, inhibition, etc)

**Neutral relationship**- This region binds basal transcriptional components such as the TATA box binding protein  [ **TBP** ]  associated factors TAFII40  [ **TAF40** ]  and TAFII60 as well as the mdm 2 and adenovirus type 5 E1B 55 kDa oncoproteins.  Ref: 9427007 Fold Des, 1997
- These results demonstrate that the TFIIA  **TAF40**  interaction is important in vivo and indicate a functional role for  **TAF40**  as a bridging factor between TFIIA and TFIID  [ **TBP** ] .  Ref: 11238911 Mol Cell Biol, 2001
- Cloning and characterization of an essential Saccharomyces cerevisiae gene,  **TAF40** , which encodes yTAFII40, an RNA polymerase II specific TATA binding protein  [ **TBP** ]  associated factor.  Ref: 9083082 J Biol Chem, 1997
- Here, a direct interaction between the activation domain of p53 and two subunits of the TFIID  [ **TBP** ]  complex, TAFII40  [ **TAF40** ]  and TAFII60, is reported.  Ref: 7809597 Science, 1995

**Non-interactive relationship** (e.g. studied together, co-existance, homology, etc.)

- We show that in addition to making direct contacts with  **TBP** , yeast  **TAF40**  interacts directly and specifically with TFIIA.  Ref: 11238911 Mol Cell Biol, 2001
- Here we show that mutations in the yeast TFIID  [ **TBP** ]  specific protein  **Taf40**  lead to a general cessation of transcription, even in the presence of excess  **TBP** , suggesting that the TFIID  [ **TBP** ]  complex is required at most promoters in vivo.  Ref: 10521393 Genes Dev, 1999
- ...  **TBP**  or a subcomplex lacking TAFII40  [ **TAF40** ]  and TAFII60 did not.  Ref: 7809597 Science, 1995
- TFIID  [ **TBP** ]  specific yeast  **TAF40**  is essential for the majority of RNA polymerase II mediated transcription in vivo.  Ref: 10521393 Genes Dev, 1999
- Here we report the molecular cloning, expression, and biochemical characterization of Drosophila TAFII40  [ **TAF40** ]  dTAFII40, a subunit of TFIID  [ **TBP** ] .  Ref: 8221891 Cell, 1993
- No significant correlation between in vivo activation function and in vitro binding to human TATA binding protein  [ **TBP** ] , human TFIIB, or Drosophila melanogaster TAFII40  [ **TAF40** ]  was observed for this set of VP16C mutants.  Ref: 9632787 Mol Cell Biol, 1998
- TFIIA interacts with TFIID  [ **TBP** ]  via association with TATA binding protein  [ **TBP** ]  and  **TAF40** .  Ref: 11238911 Mol Cell Biol, 2001
- In this report we describe the cloning and initial characterization of  **TAF40** , a gene that encodes a yeast TATA binding protein  [ **TBP** ]  associated factor yTAF of Mr = approximately 4 00.  Ref: 9083082 J Biol Chem, 1997
- However, TATA box binding protein  [ **TBP** ]   **TBP**  associated factors TAFs, or coactivators, are required for this interaction to culminate in productive transcription complex assembly, and one such TAF, Drosophila  **TAF40** , reportedly forms a ternary complex with VP16 and TFIIB.  Ref: 7597078 Proc Natl Acad Sci U S A, 1995
- The Epstein Barr virus nuclear protein 2 acidic domain can interact with TFIIB,  **TAF40** , and RPA70 but not with TATA binding protein  [ **TBP** ] .  Ref: 7983760 J Virol, 1995
- Finally, we observed that ERM bound TAFII60 via AD1 and bound  **TBP**  and TAFII40  [ **TAF40** ] , presumably via other activation domains.  Ref: 9358152 Nucleic Acids Res, 1997
